# Development of a 15-Gene Signature Model as a Prognostic Tool in Sex Hormone-Dependent Cancers

**DOI:** 10.1155/2021/3676107

**Published:** 2021-11-24

**Authors:** Zhi Xia, Jian Xiao, Aibin Liu, Qiong Chen

**Affiliations:** ^1^Department of Geriatrics, Xiangya Hospital of Central South University, Changsha, Hunan 410008, China; ^2^Department of Gerontology and Respiratory Diseases Xiangya Hospital Central South University, Changsha, Hunan 410008, China

## Abstract

Sex hormone dependence is associated with tumor progression and prognosis. Here, we explored the molecular basis of luminal A-like phenotype in sex hormone-dependent cancers. RNA-sequencing data from 8 cancer types were obtained from The Cancer Genome Atlas (TCGA). We investigated the enrichment function of differentially expressed genes (DEGs) in luminal A breast cancer (BRCA). Weighted coexpression network analysis (WGCNA) was used to identify gene modules associated with the luminal A-like phenotype, and we calculated the module's preservation in 8 cancer types. Module hub genes screened using least absolute shrinkage and selection operator (LASSO) were used to construct a gene signature model for the luminal A-like phenotype, and we assessed the model's relationship with prognosis, enriched pathways, and immune infiltration using bioinformatics approaches. Compared to other BRCA subtypes, the enrichment functions of upregulated genes in luminal A BRCA were related to hormone biological processes and receptor activity, and the downregulated genes were associated with the cell cycle and nuclear division. A gene module significantly associated with luminal A BRCA was shared by uterine corpus endometrial carcinoma (UCEC), leading to a similar phenotype. Fifteen hub genes were used to construct a gene signature model for the assessment of the luminal A-like phenotype, and the corrected *C*-statistics and Brier scores were 0.986 and 0.023, respectively. Calibration plots showed good performance, and decision curve analysis indicated a high net benefit of the model. The 15-gene signature model was associated with better overall survival in BRCA and UCEC and was characterized by downregulation of DNA replication, cell cycle and activated CD4 T cells. In conclusion, our study elucidated that BRCA and UCEC share a similar sex hormone-dependent phenotype and constructed a 15-gene signature model for use as a prognostic tool to quantify the probability of the phenotype.

## 1. Introduction

Sex steroids can directly regulate tumor progression and prognosis in a receptor-dependent manner, and these tumors are generally considered to have a sex hormone-dependent phenotype. Due to the existence of abundant sex hormone receptors, this phenotype is prominent in sex-specific cancer types originating from traditional hormone target organs, such as breast cancer (BRCA) in women or prostate adenocarcinoma (PRAD) in men [1, 2]. In addition, sex hormone-mediated actions also occur in malignancies derived from nontraditional hormone target organs, which are implicated in tumorigenesis, treatment response, and clinical outcome [2, 3]. These cancer types generally manifest significant sex-biased disparities with respect to their incidence and clinical characteristics [4], and thus, some researchers have proposed that these cancer types, at least in part, possess a sex hormone-dependent phenotype [1, 5]. A previous study investigated the influence of sex factors on these cancer types and characterized some of them as the strong sex-effect type based on their sex-affected molecular signatures [6]. However, the similarities and differences in sex hormone-dependent phenotypes among these cancer types remain poorly understood.

BRCA is a typical female sex hormone-dependent cancer, and previous studies confirmed that female sex hormones modulate tumor progression via estrogen receptors (ERs) and progesterone receptors (PRs) [7, 8]. Based on clinicopathological characteristics and immunohistochemistry for ER, PR, and human epidermal growth factor receptor 2 (HER2), BRCA is classified into four subtypes, including luminal A, luminal B, HER2-overexpressing, and triple-negative BRCA [9]. Among these subtypes, luminal A represents the sex hormone-dependent phenotype and exhibits an ideal response to endocrine therapies with a good prognosis, while the latter two subtypes have a weak relationship with sex hormones [10, 11]. Given that the gene module can be considered a relevant indication of the biological phenotype and its preservation could represent a similar phenotype [12, 13], we explored whether a gene module related to the luminal A-like phenotype exists in BRCA and assessed its preservation in other sex hormone-dependent types of cancer.

**Figure 1:**
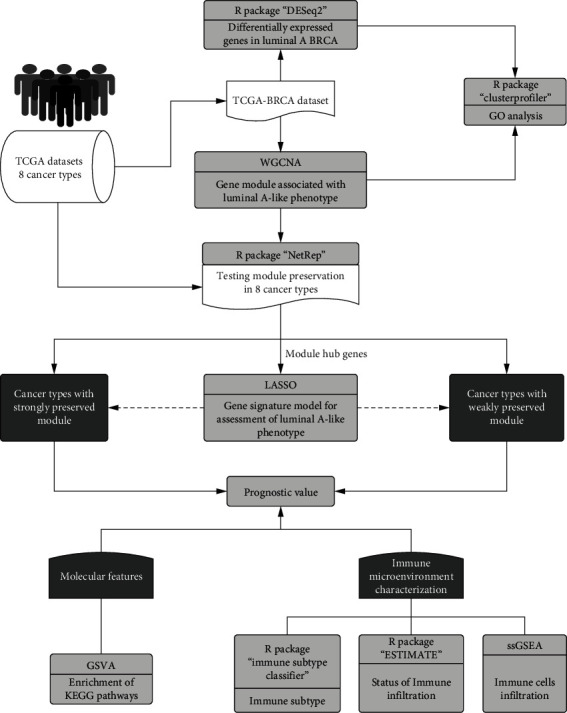
The workflow of this study. First, the gene module related to the luminal A-like phenotype was identified, and its applicability to other sex hormone-dependent cancer types was assessed. Next, module hub genes screened using LASSO were used to construct a gene signature model for the assessment of the luminal A-like phenotype. Finally, the model's relationship with prognosis, enriched pathways, and immune infiltration was investigated using bioinformatics approaches.

Using RNA sequencing (RNA-seq) data from The Cancer Genome Atlas (TCGA), the present study performed a pancancer analysis. The pipeline is illustrated in Figure 1. We performed weighted gene coexpression network analysis (WGCNA) [14] to identify the target gene module related to the luminal A-like phenotype and investigated its applicability to several sex hormone-dependent cancer types. The present study identified a 15-gene signature model that works as a prognostic tool in sex hormone-dependent cancers by quantifying the luminal A-like phenotype.

## 2. Materials and Methods

### 2.1. Data Acquisition

All mRNA expression data were obtained from TCGA (http://cancergenome.nih.gov) by utilizing cBioPortal [15]. Normalized TCGA RNA-RNA-seq v2 data (RNA-seq by expectation-maximization [16]) of BRCA (*n* = 1093), lung adenocarcinoma (LUAD, *n* = 515), lung squamous cell carcinoma (LUSC, *n* = 484), ovarian serous cystadenocarcinoma (OV, *n* = 303), uterine corpus endometrial carcinoma (UCEC, *n* = 527), bladder carcinoma (BLCA, *n* = 407), thyroid carcinoma (THCA, *n* = 501), and PRAD (*n* = 493) were used in bioinformatics and statistical analysis. We screened 261 BRCA samples with different sex hormone-dependent phenotypes: 129 samples of the luminal A subtype (ER-positive, PR-positive > 20%) and 132 samples of other BRCA subtypes (HER2 overexpressing with ER- and PR-negative or triple-negative BRCA) and acquired the preprocessing count data.

### 2.2. Identification of Differentially Expressed Genes (DEGs) and Gene Ontology (GO) Analysis

DEGs (∣Log2‐fold change | <1, *P* value < 0.05) between luminal A and other BRCA subtypes were identified using package R “DESeq2” [17]. According to the DESeq2 tutorial, we used preprocessing count data as input data for the statistical mode of “DESeq2” and extracted the top 400 genes (according to |Log2-fold change|) to create a heatmap. The R package “Clusterprofiler” [18] was applied to perform GO analysis for DEGs and visualizing outcomes. Upregulated and downregulated genes were analyzed.

### 2.3. Weighted Gene Coexpression Network Analysis (WGCNA)

We performed WGCNA for 261 BRCA samples using the R package “WGCNA” [14]. Modules identified by WGCNA were labeled using different colors to display genes sharing a similar connectivity pattern. Gene expression profiles within each module are summarized as a summary expression, also called the first principal component or module eigengene. The biological functions of the target module that were significantly associated with luminal A BRCA were explored using GO analysis, and hub genes were key elements in the module and comprised the central point of the network architecture. Gene significance (GS) is the correlation between the expression profile and clinical traits. Module membership (MM) is the relevance of the expression profile to each module eigengene. Module hub genes were defined as those with GS > 0.2 and MM > 0.8.

### 2.4. Assessing the Preservation of Network Modules

Module preservation analysis is used to quantify the replication of target module, and the outcome could be interpreted as an indicator of biological relevance [12, 13].We used the R package “NetRep” for statistically testing replication and preservation of the target module, which computes module preservation from seven statistics [19]. We used WGCNA to obtain the soft-thresholding power as input data for ‘NetRep' to obtain matrix containing the network edge weights encoding the interaction strength between each pair of genes. Another 832 BRCA samples were used as validation cohort to assess the preservation of the target module in BRCA. According to the tutorial, a module was considered strongly preserved if the *P* value was <0.01 for all seven preservation statistics, weakly preserved if one or more, but not all, test statistics were *P* < 0.01, and no evidence if no test statistics were *P* < 0.01.

### 2.5. Development and Validation of a Gene Signature Model

We regarded hub genes that were also DEGs as candidate variables for the least absolute shrinkage and selection operator (LASSO) [20] and used screened hub genes to construct a gene signature model using multiple logistic regression. The model can transform the predictive value for the luminal A-like phenotype into a continuous variable, which provides an advantage during subsequent statistical analyses. Brier scores were used to calculate the performance of the gene signature model, and lower scores indicated increased predictive accuracy. Predictive power was measured by the area under the receiver operating characteristic curve, also called the concordance index (namely, the *C*-statistic), and bootstrapping validation using 100 resamples was conducted to calculate the corrected value [21]. The calibration curve provided a comparison between the expected and observed conversion probabilities. To assess the clinical utility of the nomogram developed in the present study, decision curve analysis (DCA) [22] was conducted. The DCA plot displays the net benefit of model-based decisions at different threshold probabilities, and three DCA curves present cases with model predicting outcome, all cases with the outcome, and no cases with the outcome. The “glmnet,” “rms,” “pROC,” and “dca” packages in R were used in the process.

### 2.6. Gene Set Variation Analysis (GSVA)

We utilized the R package “GSVA” to perform GSVA, which implements a nonparametric unsupervised method and quantifies the enrichment of gene sets. The 50-gene analysis of microarrays for risk of recurrence (PAM50-ROR) is a well-validated gene-based signature assay available for assessing the luminal A-like phenotype [23]. We customized the 50-gene set as input object for GSVA to assess its enrichment score in luminal A BRCA. According to the predicted probabilities of the gene signature model, samples were separated into high (>50%) and low luminal A-like phenotype groups (≤50%), and GSVA was further used to calculate the enrichment score of pathways in different groups [24]. *P* < 0.05 was regarded as statistically significant. The gene set “c2.cp.kegg.v7.0.entrez.gmt,” downloaded from the Molecular Signature Database (MSigDB), was regarded as the reference gene set for pathway analysis.

### 2.7. Validating Prognostic Value of Model

The R packages “survminer” and “survival” were used to evaluate the prognostic value of the gene signature model for patient overall survival (OS). Patients were divided into two groups based on the predicted probabilities of the gene signature model to plot the Kaplan-Meier survival curves.

### 2.8. Immune Microenvironment Characterization

Several bioinformatics tools were used to identify the immune characteristics of the luminal A-like phenotype. The R package “ImmuneSubtypeClassifier” was used to characterize immune subtypes in cancer types, including wound healing, IFN-*γ* dominant, inflammatory, lymphocyte depleted, immunologically quiet, and TGF-*β* dominant, which are associated with tumor prognosis and clinicopathological features [25]. In addition, the R package “ESTIMATE” was used to reflect the number of tumor-infiltrating immune cells [26]. Moreover, we used the R package “GSVA” to perform a single-sample gene set enrichment analysis (ssGSEA) to identify the significant immune cells related to the luminal A-like phenotype based on metagenes of 28 immune cells related to adaptive and innate immune systems, whose expression has been shown to accurately predict the infiltration of immune cell populations [27].

### 2.9. Statistical Analysis

R statistical software (v.3.6.1) was used for statistical analysis and graphical visualization. The null hypotheses were rejected at *P* values lower than 0.05. Analysis was performed on log2-transformed gene expression. A Spearman correlation analysis was used to estimate the correlation between the linear predictors of the gene signature model (probabilities on logit scale) and the enrichment score of the PAM50-gene signature. The Wilcoxon test was used to compare the distributions of two sets of any continuous variable. The influence of the immune cell on survival was calculated using the Cox regression model.

## 3. Results

### 3.1. Identification Enrichment Functions of DEGs

**Figure 2:**
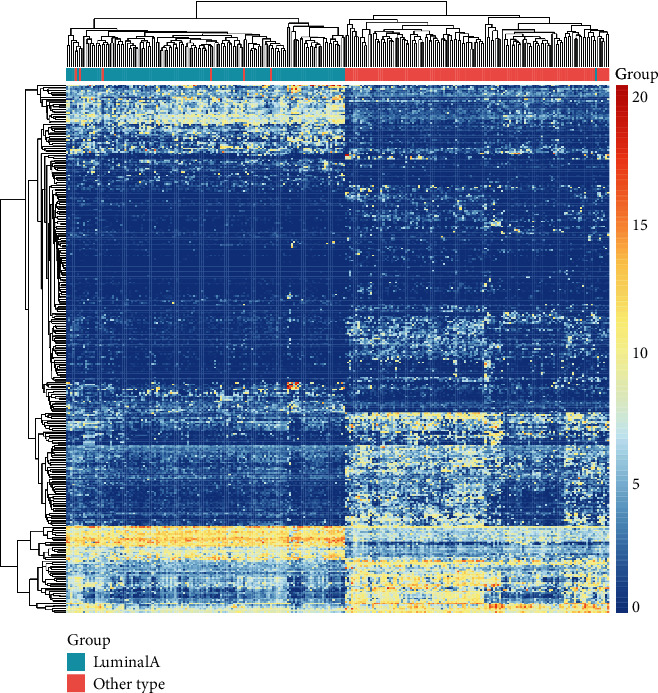
Heatmap of the top 400 differentially expressed genes between luminal A and other subtypes of BRCA (HER2 overexpressing with ER- and PR-negative or triple-negative BRCA).

**Figure 3:**
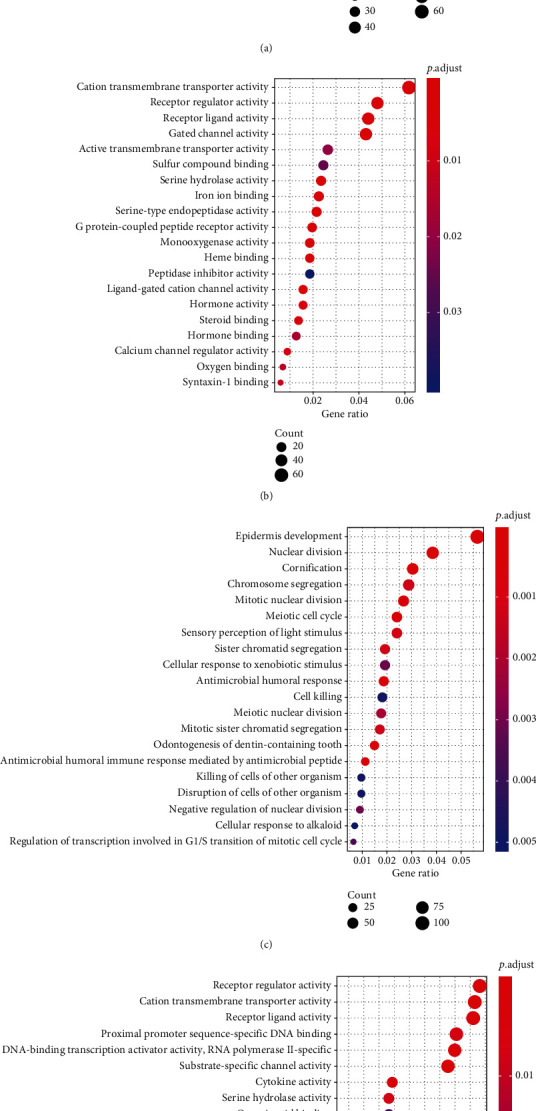
The enrichment function of differentially expressed genes. Bubble diagram showing the (a) biological process and (b) molecular function of upregulated genes. (c) Biological process and (d) molecular function of downregulated genes.

A total of 3543 genes were identified as DEGs between luminal A and other BRCA subtypes (HER2 overexpressing with ER- and PR-negative or triple-negative BRCA), including 1324 upregulated and 2219 downregulated DEGs. The heatmap of the top 400 genes (according to |Log2-fold change|) is shown in Figure 2, revealing the different patterns of genomic expression profiles between luminal A BRCA and other subtypes. GO analysis for the upregulated and downregulated DEGs was performed (Figure 3). For upregulated genes, signal release, hormone-related biological process, and receptor activity were primarily enriched (Figures 3(a) and 3(b)), and for downregulated genes, cell cycle and nuclear division were primarily enriched (Figures 3(c) and 3(d)).

### 3.2. Assessment of the Preservation of Gene Modules Related to the Luminal A-Like Phenotype

**Figure 4:**
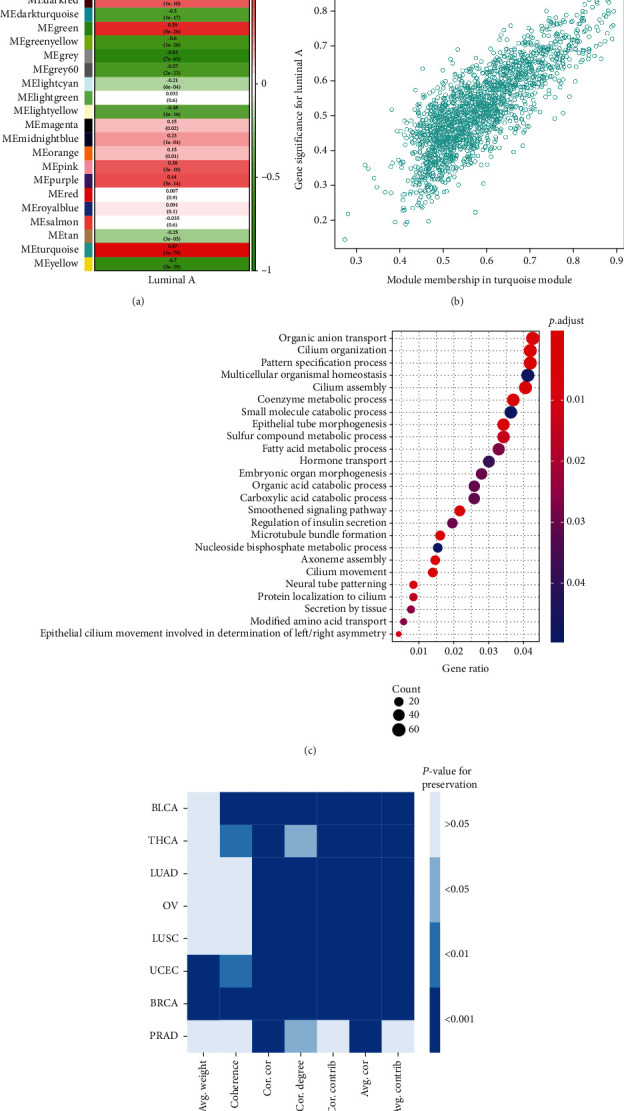
Weighted gene coexpression network analysis of luminal A versus other subtypes of BRCA (HER2 overexpressing with ER- and PR-negative or triple-negative BRCA). (a) The correlation between gene modules and luminal A BRCA. (b) The correlation between module membership and gene significance in the turquoise module. (c) Enriched gene ontology functions of the turquoise module. (d) Assessment of the preservation of the turquoise module. The statistical significance of seven module preservation statistics for 8 types of cancer. The module is considered to be preserved if all statistics had a permutation test *P* value < 0.01.

The weighted gene coexpression network of BRCA was identified into 27 gene modules, and Figure 4(a) summarizes the correlation between gene modules and luminal A BRCA. The turquoise module was significantly associated with luminal A BRCA (Figure 4(b)). GO analysis revealed that the module is related to multiple metabolic and secretory processes involving hormones (Figure 4(c)). The preservation analysis revealed that the gene module is strong preserved in BRCA and UCEC but weakly preserved in other cancer types, including OV, PRCA, LUAD, LUSC, THCA, and BLCA (Figure 4(d)).

### 3.3. Developing and Validating a 15-Gene Signature Model

**Figure 5:**
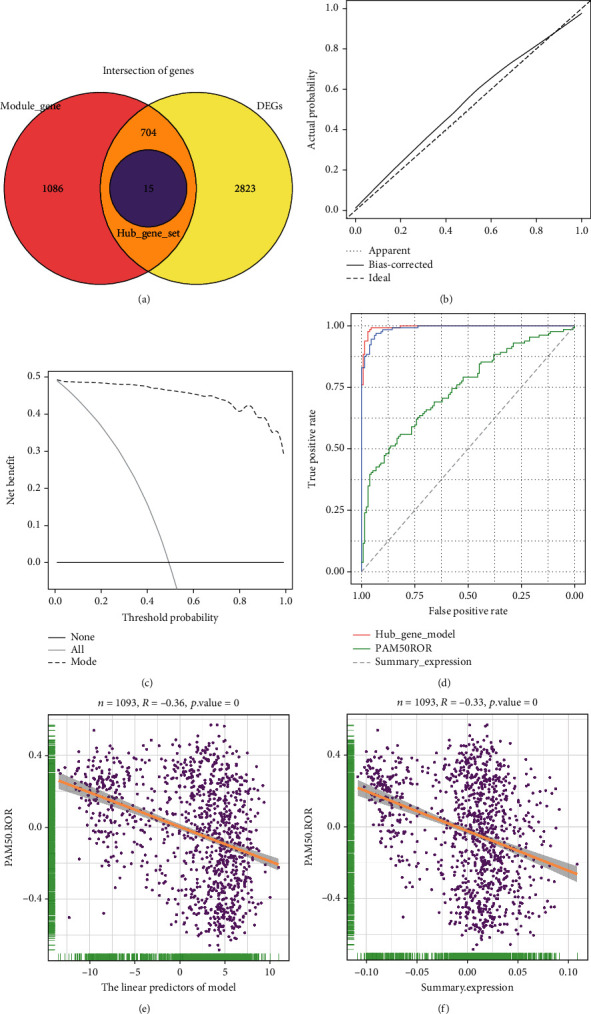
Construction and evaluation of the 15-gene signature model. (a) The intersection of genes between the turquoise module and differentially expressed genes. (b) Calibration curve, (c) decision curve analysis, and (d) receiver operating characteristic curve of the 15-gene signature model. Scatter plots showing the correlation between the enrichment score of PAM50-gene signature and (e) the linear predictors of the 15-gene signature model or (f) the turquoise module's summary expression in BRCA. 0 indicates *P* < 0.01.

A total of 704 common genes existed in the turquoise module and DEGs, and 56 genes were defined as hub genes. Fifteen hub genes were screened using LASSO: EFCAB12, AGR3, ANXA9, CFAP61, DEGS2, ESR1, FSIP1, C5AR2, KCNJ11, KDM4B, PGR, SCUBE2, SLC7A8, THSD4, and TTC8 (Figure 5(a)). To make subsequent evaluation of the luminal A phenotype more convenient, a 15-gene signature model was established, and detailed information on the model can be found in Supplementary Material Table S1. In the 261 BRCA cohort, the corrected *C*-statistic and the Brier scores were 0.986 and 0.023, respectively, suggesting that the model had good discriminative ability. The calibration plots of the 15-gene signature model showed that the agreement between the predicted and observed situations was optimal (Figure 5(b)), and DCA demonstrated that the model conveys a significant net benefit (Figure 5(c)), demonstrating the potential application value of the 15-gene signature model in the assessment of the luminal A-like phenotype. Compared to the predictive value of the PAM50-gene signature, both the 15-gene signature model and turquoise module summary expression exhibited better performance in the assessment of the luminal A-like phenotype (Figure 5(d)). Although the 15-gene signature model had a nearly identical area under the curve as the turquoise module's summary expression, it was simpler to use for calculating the luminal A-like phenotype.

We then tested the correlation between the linear predictors of the 15-gene signature model and the enrichment score of the PAM50-gene signature, and the PAM50-gene signature exhibited a significant negative correlation with the linear predictors in BRCA (Figure 5(e)). A consistent correlation was observed between the module's summary expression and the enrichment score of the PAM50-gene signature (Figure 5(f)).

### 3.4. Validation of the Model's Prognostic Value

**Figure 6:**
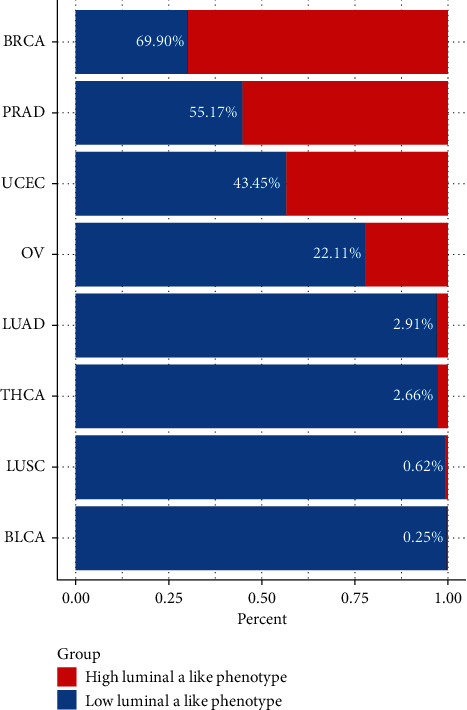
The distribution of tumor samples with a high or low luminal A-like phenotype in different types of cancer.

**Figure 7:**
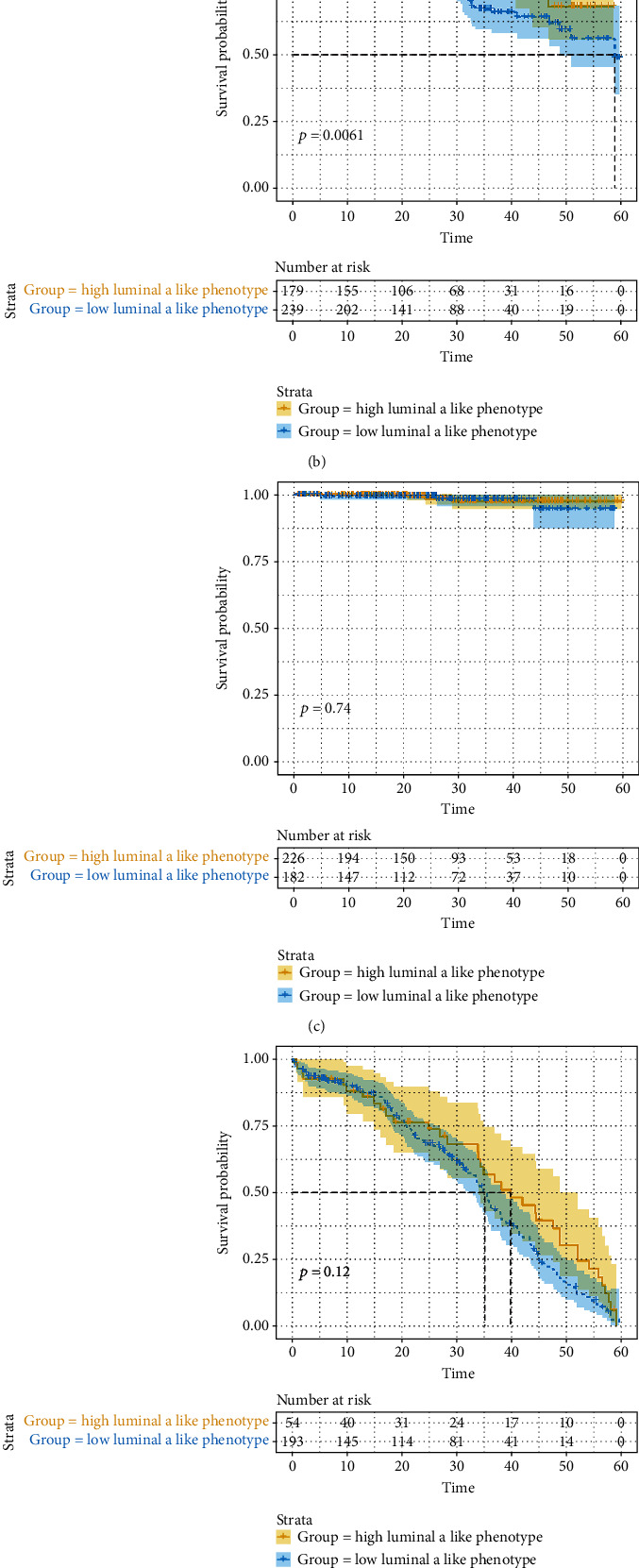
Survival analysis for overall survival (OS) time in the (a) TCGA-BRCA, (b) TCGA-UCEC, (c) TCGA-PRAD, and (d) TCGA-OV datasets. The yellow line indicates samples with a high luminal A-like phenotype, and the blue line designates samples with a low luminal A-like phenotype. Violin plots showing the GSVA enrichment scores of (e) cell cycle and (f) DNA replication pathways in BRCA, OV, PRAD. and UCEC samples with a high or low luminal A-like phenotype. ^∗∗^*P* < 0.01, ^∗∗∗∗^*P* < 0.0001.

We investigated the distribution of the luminal A-like phenotype in multiple cancer types based on the model and found that up to 69.9% of BRCA samples were characterized as the high luminal A-like phenotype group, and the proportion of the high luminal A-like phenotype gradually shrank in PRAD, UCEC, and OV samples, while few samples of LUAD, THCA, LUSC, and BLCA were judged as having a high luminal A-like phenotype (Figure 6). Given the limited samples of LUAD, THCA, LUSC, and BLCA, we further explored the prognostic value of the 15-gene signature model in the BRCA, UCEC, PRAD, and OV cohorts and investigated the difference between cancer types with the weak or strong preserved target module. Compared to the low luminal A-like phenotype group, the high luminal A-like phenotype group displayed a longer OS in BRCA patients (Figure 7(a)), and the same result was observed in UCEC patients (Figure 7(b)). However, the luminal A-like phenotype was not associated with a survival benefit for PRCA or OV patients (Figures 7(c) and 7(d)). We further explored the potential molecular mechanism behind this outcome, and GSVA revealed that signaling pathways involved in the cell cycle and DNA replication were significantly downregulated in the high luminal A-like phenotype group in both BRCA and UECE, while the same result was observed in PRAD and OV (Figures 7(e) and 7(f)).

### 3.5. Immune Characterization of the Luminal A-Like Phenotype

**Figure 8:**
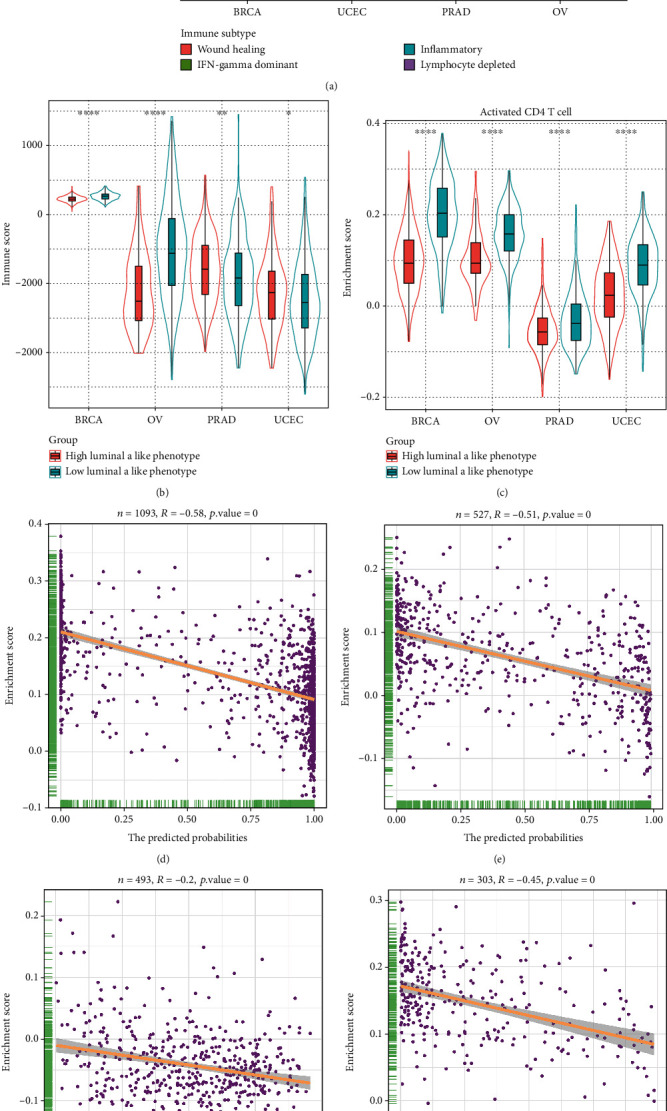
Immune subtypes and infiltrating immune cells related to the luminal A-like phenotype. (a) The pie matrix showing 4 immune subtypes identified in BRCA, UCEC, PRAD, and OV samples with a high or low luminal A-like phenotype. Violin plots showing (b) immune scores and (c) enrichment scores of activated CD4 T cells in BRCA, OV, PRAD, and UCEC samples with a high or low luminal A-like phenotype. ^∗^*P* < 0.05, ^∗∗^*P* < 0.01, and ^∗∗∗∗^*P* < 0.0001. Scatter plots showing the correlation between the enrichment score of activated CD4 T cells and the predicted probabilities of a luminal A-like phenotype for (d) BRCA, (e) UCEC, (f) PRAD, and (g) OV samples. 0 indicates *P* < 0.01.

Immune cells influence tumor growth and clinical outcome via cytotoxicity and cytokines, and thus, we assessed the relationship between the luminal A-like phenotype and immune infiltration. We found that 4 main immune subtypes comprise these cancer types: the wound healing and the IFN-*γ* dominant types, which possesses a high proliferation rate; the inflammatory type conveys a low to moderate tumor cell proliferation and has the best prognosis; and the lymphocyte-depleted type is characterized as having the least favorable outcome. We found that the wound healing type was the dominant immune subtype in both BRCA and UCEC. Compared to samples defined as having a low luminal A-like phenotype, the proportion of inflammatory subtype increases, while the IFN-*γ* dominant type decreases in the group with a high luminal A-like phenotype. Different patterns of immune subtypes exist in PRAD and OV, and the inflammatory type or lymphocyte-depleted type dominates their high luminal A-like group (Figure 8(a)). ESTIMATE immune scores revealed a different status of infiltrating immune cells between high or low luminal A-like samples in these 4 types of cancer (Figure 8(b)). Further analysis demonstrated that a majority of immune cells exhibit a varying infiltration pattern, while activated CD4 T cells are an intriguing exception and were consistently decreased in tumor samples with a high luminal A-like phenotype (Figure 8(c), Supplementary Material Figure S1). Correlation analysis revealed a significant negative correlation between activated CD4 T cell enrichment and the predicted probabilities of the luminal A-like phenotype (Figures 8(d)–8(g)): Spearman's correlation coefficients were -0.58, -0.51, and -0.45 in BRCA, UCEC, and OV, respectively, and -0.2 in PRAD. A univariate Cox regression model revealed that activated CD4 T cells did not influence overall survival (Supplementary Material Table S2).

## 4. Discussion

In this study, we applied bioinformatics approaches to identify a gene module associated with the luminal A-like phenotype and assessed its replication in several sex hormone-dependent types of cancer [6]. We identified a common gene module between BRCA and UCEC and constructed a 15-gene signature model that could quantify the probabilities of a luminal A-like phenotype and work as a prognostic tool.

The obvious distinctions in the phenotype of BRCA subtypes provide a clue to investigate the molecular basis of sex hormone dependence. Compared to other BRCA subtypes, a different gene expression pattern exists in luminal A BRCA, which is related to the downregulation of the cell cycle and nuclear division. This could explain the low proliferation rate and favorable prognosis of luminal A BRCA. In addition, we demonstrated that the target gene module was strongly preserved in UCEC, a female sex hormone-dependent cancer, and patients can benefit from antifemale sex hormone therapies [1]. Other types of cancer possess a weakly preserved module. Similar to BRCA and UCEC, OV is a female-specific cancer, and the specificity of tissue might make a difference in the female sex hormone-dependent phenotype. PRAD and BLCA are primarily related to the regulation of androgens and their receptors [28–30], and inhibiting androgen receptor signaling and androgen deprivation therapy are well established to restrain tumor cell biological behaviors and provide patients with benefits in prolonging the recurrence period [31, 32]. We presumed that the distinct molecular differences between male and female sex hormone dependence made the difference. THCA and LUAD occur more frequently in women, but the role of sex hormones is still controversial, and studies have demonstrated that female sex hormones have little impact on outcome [33, 34]. In LUSC, smoking and exposure to secondhand smoke rather than sex hormones play a major role in the etiology [35, 36]. In these cancer types, female sex hormones are inferior influencing factors.

Genomic expression profiles can determine the molecular phenotype [37], so it is reasonable that UCEC might share a similar phenotype with BRCA. We constructed a 15-gene signature model using selected hub genes in the target module, and this model exhibited good performance in qualifying the probabilities of a luminal A-like phenotype in BRCA. We confirmed that both the module's summary expression and the linear predictors of the 15-gene signature model were negatively correlated with the PAM50-gene signature, a well-validated prognostic tool for BRCA, and a low PAM50-ROR for luminal A subtype was confirmed [23, 38]. We found that BRCA and UCEC patients with a high luminal A-like phenotype had a longer OS. This result is consistent with the fact that the luminal A-like phenotype has a better prognosis. The outcome supports the gene signature model, which is derived from the replicated gene module, be used as a predictive tool for calculating the luminal A-like phenotype. In addition, the 15-gene signature model had a larger area under the curve than the PAM50-gene signature, suggesting improved efficacy for predicting outcome. Regarding PRAD and OV, the high luminal A-like phenotype conveyed no survival benefit. However, downregulated pathways related to the cell cycle and DNA replication were observed in BRCA, UCEC, PRAD, and OV samples with a high luminal A-like phenotype, and this is more suggestive of other factors influencing the clinical outcome of the luminal A-like phenotype. The tumor immune microenvironment, which has a relationship with patient prognosis [39], has come into focus.

A previous study reported that the wound healing type, an immune subtype related to proliferation ability, is predominant in luminal A BRCA [25], which is a consistent outcome observed in the present study. At the same time, BRCA and UCEC samples with a high luminal A-like phenotype evince a decreased proportion of the highly proliferative subtype accompanying a raised proportion of lower proliferative subtype, while a different proportion of immune subtypes was composed of PRAD and OV. In addition, a varying status of infiltrating immune cells in these 4 types of cancer was revealed. The result was in agreement with a previous study, and the heterogeneity of immune infiltration patterns was identified in solid tumors, including BRCA, PRAD, UCEC, and OV [40]. Among 28 subpopulations of infiltrating immune cells, we found that activated CD4 T cells have a significantly negative correlation with the luminal A-like phenotype, but they bring no benefits to patient prognosis. A previous study revealed that ER-positive BRCA without immune infiltration exhibits a similar clinical outcome to ER-negative BRCA with high infiltration, irrespective of cell type, and activated CD4 T cells have no impact on the clinical outcome of BRCA [39]. Consistent with this study, Chen et al. found that a high level of infiltrating immune cells brings luminal A BRCA patients no prognostic benefit but is associated with a shorter disease-free survival in Western patients [41]. These studies suggest that tumor molecular subtype and intratumoral immune cell infiltration have an identical prognostic influence. Teschendorff et al. proposed that the prognosis of ER-positive BRCA is related to the status of mitotic cell cycle functions, whereas the outcome of ER-negative BRCA is associated with the immune response [42]. Therefore, the survival benefits of a high luminal A-like phenotype might primarily originate from intrinsic tumor molecular features, including sex hormone receptors, rather than the extrinsic factor of immune infiltration [43]. However, the immune microenvironment might have major implications for the clinical outcome of PRAD and OV. The inflammatory subtype has the best prognosis and is the dominant immune subtype in PRAD, which conveys a prognostic benefit. The immune subtype of OV is characterized by increased intratumor heterogeneity and the least favorable outcome, which might counteract the benefits of the luminal A-like phenotype [25, 44]. The tumor immunity in cooperation with molecular subtype exerts fundamental differences in the patterns of patient prognosis, that is, the differences in the luminal A-like phenotype among these types of cancer.

The luminal A-like phenotype might be a specific subtype in most sex hormone-dependent cancers. We confirmed that a similar molecular basis between BRCA and UCEC, and the 15-gene signature model is applicable for predicting their prognosis. Except for PAM50-ROR, the 21-gene recurrence score is another classical gene signature used as a prognostic assay for BRCA, and patients with recurrence score < 10 were most likely the luminal A subtype [45], but no research has shown these 2 gene signatures can be applied in UCEC. Furthermore, as a positive correlation index, the 15-gene signature model is more convenient to apply.

Some limitations exist in the present study. First, our study was based on datasets from TCGA, and the batch effect is evident, although we used log2-transformed normalized-data to minimize this effect. Second, we lacked sufficient information to externally validate the 15-gene signature model in UCEC cohort. The clinical utility of the 15-gene signature will be explored in future studies.

## 5. Conclusions

In summary, this study elucidated that BRCA and UCEC share a luminal A-like phenotype. We constructed a 15-gene signature model to quantify the probability of the sex hormone-dependent phenotype that can be used as a prognostic tool in BRCA and UCEC patients.

## Data Availability

All the RNA-seq data can be downloaded from the TCGA dataset: https://www.cancer.gov/tcga.

## Supplementary Materials

Supplementary Materials Table S1: multivariate logistic regression analysis of 15 hub genes. Table S2: univariate Cox regression analysis of activated CD4 T cells for patient prognosis. Figure S1: the correlation between the luminal A-like phenotype and infiltrating immune cells in tumor samples.
