# Supplementary material for: Development of a 15-Gene Signature Model as a Prognostic Tool in Sex Hormone-Dependent Cancers
Source: Biomed Res Int. 2021 Nov 24;2021:3676107. doi: 10.1155/2021/3676107 (PMC8635877; doi:10.1155/2021/3676107)
Supplement: Supplementary Materials — Table S1: multivariate logistic regression analysis of 15 hub genes. Table S2: univariate Cox regression analysis of activated CD4 T cells for patient prognosis. Figure S1: the correlation between the luminal A-like phenotype and infiltrating immune cells in tumor samples. [file 3676107.f1.zip › Supplementary_Figure S1.pdf]

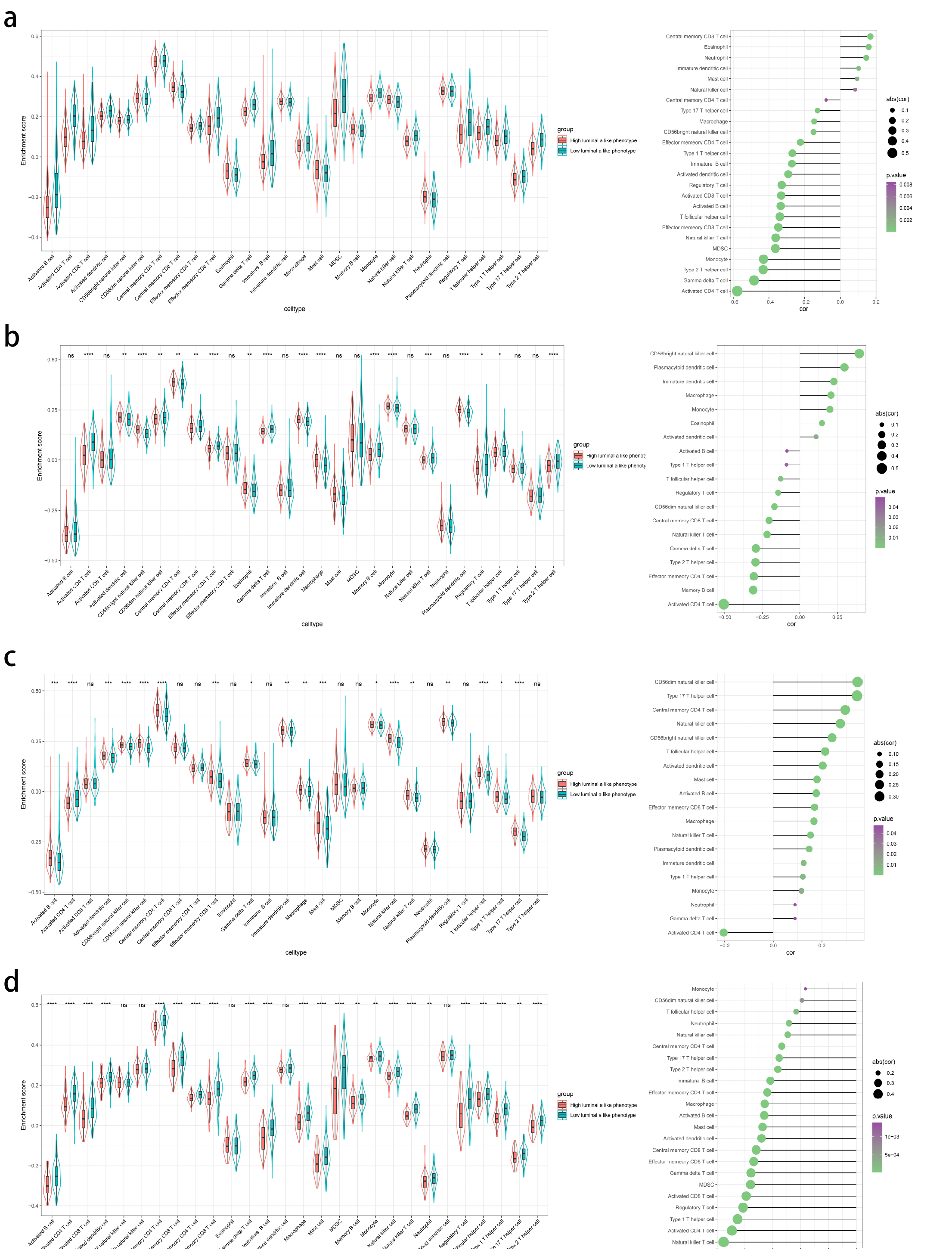

Figure S1: The correlation between the luminal A-like phenotype and infiltrating immune cells in tumor samples. Violin plot showing the enrichment score of 28 infiltrating immune cells in (a) BRCA, (b) UCEC, (c) PRAD and (d) OV samples with a high or low luminal A-like phenotype, and bubble diagram showing their correlation with the predicted probabilities of a luminal A-like phenotype. ns indicates p ⩾ 0.05, *p <0.05, ** p <0.01, ***p <0.001, ****p <0.0001.
