# Supplementary material for: Development of a 15-Gene Signature Model as a Prognostic Tool in Sex Hormone-Dependent Cancers
Source: Biomed Res Int. 2021 Nov 24;2021:3676107. doi: 10.1155/2021/3676107 (PMC8635877; doi:10.1155/2021/3676107)
Supplement: Supplementary Materials — Table S1: multivariate logistic regression analysis of 15 hub genes. Table S2: univariate Cox regression analysis of activated CD4 T cells for patient prognosis. Figure S1: the correlation between the luminal A-like phenotype and infiltrating immune cells in tumor samples. [file 3676107.f1.zip › Supplementary_TableS1.pdf]

Table S1: Multivariate Logistic Regression Analysis of 15 hub genes

Gene	OR	95%CI	P value
EFCAB12	2.171149e+00	8.355723e-01 - 6.209669e+00	0.11821
AGR3	1.043819e+00	6.312404e-01 - 1.727068e+00	0.86184
ANXA9	1.103383e+00	5.958385e-01 - 2.098362e+00	0.75186
CFAP61	1.248300e+00	5.668866e-01 - 3.109327e+00	0.59580
DEGS2	1.339750e+00	6.189079e-01 - 2.907962e+00	0.44913
ESR1	1.376902e+00	7.248594e-01 - 2.980942e+00	0.35585
FSIP1	1.047423e+00	5.890971e-01 - 1.749244e+00	0.86299
C5AR2	1.002159e+00	4.241985e-01 - 2.457234e+00	0.99609
KCNJ11	9.915734e-01	3.886991e-01 - 2.535844e+00	0.98571
KDM4B	1.322171e+00	3.624411e-01 - 4.909236e+00	0.66997
PGR	1.127002e+00	6.060668e-01 - 2.030695e+00	0.68744
SCUBE2	1.333304e+00	7.726794e-01 - 2.286714e+00	0.29249
SLC7A8	1.457315e+00	4.575108e-01 - 5.050388e+00	0.52923
THSD4	1.037953e+00	3.648108e-01 - 2.906070e+00	0.94272
TTC8	1.577199e+00	2.809973e-01 - 1.129709e+01	0.61598
