# Supplementary material for: Development of a 15-Gene Signature Model as a Prognostic Tool in Sex Hormone-Dependent Cancers
Source: Biomed Res Int. 2021 Nov 24;2021:3676107. doi: 10.1155/2021/3676107 (PMC8635877; doi:10.1155/2021/3676107)
Supplement: Supplementary Materials — Table S1: multivariate logistic regression analysis of 15 hub genes. Table S2: univariate Cox regression analysis of activated CD4 T cells for patient prognosis. Figure S1: the correlation between the luminal A-like phenotype and infiltrating immune cells in tumor samples. [file 3676107.f1.zip › Supplementary_TableS2.pdf]

Table S2: Univariate Cox regression analysis of activated CD4 T cells for patient prognosis

Cancer type	Coefficient	Hazard Ratio (95% CI)	P value
BRCA	-0.433125525	0.648479087 (0.108600948-3.872204941)	0.634743
UCEC	1.524261351	4.591750624 (0.270000615-78.08935462)	0.291739
PRAD	5.059762412	157.5530792 (0.011076469-2241054.661)	0.299715
OV	0.004406198	1.004415919 (0.091289103-11.05116932)	0.997127

BRCA: breast cancer; OV: ovarian serous cystadenocarcinoma; PRAD: prostate adenocarcinoma; UCEC: uterine corpus endometrial carcinoma.
